# Hypoglycemic Activity and Antioxidative Stress of Extracts and Corymbiferin from *Swertia bimaculata In Vitro* and *In Vivo*

**DOI:** 10.1155/2013/125416

**Published:** 2013-10-24

**Authors:** Zhaoxia Liu, Luosheng Wan, Yuedong Yue, Zuoqi Xiao, Yutang Zhang, Yonglong Wang, Cuiping Chen, Qiuxia Min, Jiachun Chen

**Affiliations:** ^1^Hubei Key Laboratory of Natural Medicinal Chemistry and Resource Evaluation, Tongji School of Pharmaceutical Sciences, Huazhong University of Science and Technology, Wuhan 430030, China; ^2^Hubei Key Laboratory of Natural Products Research and Development, College of Chemistry and Life Science, China Three Gorges University, Yichang 443002, China

## Abstract

The present study was to investigate the anti-diabetic activities of *Swertia bimaculata*. Based on the glucose consumption of *S. bimaculata *extractsand different fractions (petroleum, dichloromethane, ethyl acetate, *n*-butanol and water extracts) in 3T3-L1 adipocyte assay, ethanol (ETH) and dichloromethane (DTH) extracts had the most effective potency. Furthermore, ETH, DTH and corymbiferin (the most abundant component of DTH) were evaluated for anti-diabetic effects in high fat and sucrose fed combined with low dose streptozocin induced diabetic rats. DTH and corymbiferin displayed remarkable anti-diabetic activities. The fasting blood glucose levels were significantly decreased, while the serum insulin levels were obviously increased. The oral glucose tolerance was also improved. The lowed serum total cholesterol, low density lipoprotein (LDL) and triglyceride levels and increased ratio of HDL (high density lipoprotein)/LDL were observed. The insulin sensitivity was improved on the basis of increased expressions of insulin-receptor substrate-2, phosphatidylinositol 3-kinase and Ser/Thr kinase AKT2. And also DTH and corymbiferin improved antioxidant capacity and carbohydrate metabolism in diabetic rats, along with the improvement of histopathology of livers and pancreatic **β** cells. Corymbiferin was one of active constituents, responsible for anti-diabetic properties. Therefore, *S. bimaculata* could be considered as an alternative agent against diabetes mellitus.

## 1. Introduction

Diabetes mellitus (DM) characterized by hyperglycemia is a metabolic disorder of carbohydrate, fat, and protein, as a consequence of relative or absolute deficiency of insulin secretion, insulin action, or both [1]. Hyperglycemia is known to be one of the main contributors to oxidative stress by the direct generation of excessive reactive oxygen species (ROS), resulting from an imbalance between antioxidants and oxidants [2–5]. ROS can be transformed into the more potent hydroxyl radical, resulting in peroxidation of membrane lipids and damage of DNA and proteins [6–8]. ROS also play a central role in the progression of microvascular (retinopathy, neuropathy, and nephropathy) and macrovascular (heart attack, stroke, and peripheral vascular disease) complications in diabetes [9, 10]. Though insulin and other synthetic antidiabetic drugs are available, continuous treatment with them may cause undesirable adverse effects [11]. For this reason, there is a growing interest in developing traditional medicinal herbs or natural products as safer complementary and alternative agents with higher achievable antidiabetic activities [12]. The agents possessing antidiabetic effects accompanied with antioxidant activities are also beneficial for DM and its complications.

As one of these potent resources, the *Swertia* genus plants with their antidiabetic ability have attracted great attention in East Asian countries. In ayurvedic traditional medicine, the whole plants of* Swertia chirayita* are used to treat hepatitis, febrifuges, ulcer, and diabetes [13, 14]. In more modern medical usage, it is found that the preparations of *Swertia japonica* [15] and its compounds, such as swerchirin and bellidifolin [16–19], can alleviate the hyperglycemic status in diabetic animals. And also *Swertia bimaculata,* widely distributed in most parts of Asia, has long been used for the treatment of dyspepsia, urinary infection, and hepatitis diseases in middle-western ethnic district of China [20, 21]. In recent studies, sixteen xanthone compounds, which were structurally similar to these components of *Swertia japonica*, have been isolated from *S. bimaculata* [22, 23]. However, there is still no direct modern report on the antidiabetic activities of *S. bimaculata*. Therefore, this present study was carried out to investigate the antidiabetic substances of *S. bimaculata *plant and its possible mechanism(s) using *in vitro* and *in vivo* models, with a view to ascertain the use of this plant in controlling diabetes.

## 2. Materials and Methods

### 2.1. Chemicals and Reagents

Streptozotocin (STZ) was purchased from Sigma-Aldrich (St. Louis, MO, USA). The kits were indicated under specific sections. All other chemical reagents used in the study were of the highest analytical or chromatographic grade.

### 2.2. Plant Material and Preparation of Extracts

The whole herbs of *S. bimaculata* were acquired from Enshi, Hubei, China, in October 2010 and authenticated by Professor Jiachun Chen, Tongji School of Pharmaceutical Sciences, Huazhong University of Science and Technology. The voucher specimen (no. 20101012) was deposited in Hubei Key Laboratory of Natural Medicinal Chemistry and Resource Evaluation, Huazhong University of Science and Technology. The air-dried whole plants were milled and refluxed with 90% ethanol twice (1 : 10, w/v) for 3 h each time at 65°C. After filtration, the solution was combined and concentrated to obtain the crude extract (yield 24.5% relative to crude medicines) used as ETH extract. The ETH extract was suspended in water and partitioned successively to obtain petroleum (PET, yield 1.7%), dichloromethane (DTH, yield 2.5%), ethyl acetate (EAT, yield 0.4%), *n*-butanol (BUT, yield 3.8%), and water extracts. Three xanthone compounds were obtained from DTH extract by a silica gel column and identified by comparison of ^13^C-NMR spectra data in the literature [23–25]. The three compounds were used as chemical markers for HPLC analysis. All extracts and corymbiferin of the above three compounds were used for antidiabetic assay, respectively. 

### 2.3. HPLC Analysis

ETH and DTH extracts were, respectively, dissolved in methanol to the desired concentration of 1 mg dry extract/mL. After being filtered (0.22 *μ*m, Millipore, USA), a 10 *μ*L aliquot was injected into DIONEX ultimate 3000 HPLC instrument (DIONEX, USA) consisting of an autosampler and a DAD detector. Liquid chromatographic separation was achieved on a Kromasil C18 column (4.6 × 250 mm, 5 m, Sweden), using solvent A (methanol) and solvent B (0.1% formic acid aqueous), with a flow rate of 0.8 mL/min and detection at 254 nm. The gradient program was as follows: 0–20 min, 20–40% A; 20–25 min, 40–50% A; 25–45 min, 50–70% A; 45–60 min, 70–90% A. Standard substances were prepared in our laboratory as illustrated above. 

### 2.4. Preliminary Screening of Glucose Consumption of Extracts on 3T3-L1 Cells

3T3-L1 cells were differentiated as described previously [26, 27] with minor modifications. Briefly, confluent preadipocytes were cultured for 2 days in Dulbecco's modified Eagle's medium (DMEM, Gibco, USA) containing 25 mM glucose, 10% fetal bovine serum (FBS, Gibco, USA) further supplemented with 5 mg/L insulin (Sigma, St. Louis, MO, USA), 0.5 mM isobutylmethylxanthine (Sigma, St. Louis, MO, USA), and 5 *μ*M dexamethasone (Sigma, St. Louis, MO, USA) and an additional 2 days in culture medium with 5 mg/L insulin. After another 4–8 days in normal medium, about 90% of the cells showed accumulation of fat droplets. The cells were treated with the ETH, PET, DTH, EAT, BUT, water extracts, and positive metformin. All treatments were carried out after serum starvation. After 24 h, the media were collected for the measure of glucose consumption by GOD-POD method (Jiancheng, Nanjing, China). 

### 2.5. Animals and Treatment

Male Wistar rats, initially weighing 110 ± 10 g, were purchased from the Laboratory Animal Institute of Hubei Disease Control Center, China (Reg. no. SCXK (Hubei) 2008-0005). The rights of experimental animals were ensured quantum satis during the experiment. Rats were housed in constant conditions at a temperature of 24 ± 2°C, humidity of 60 ± 5%, and on a 12 h dark/light cycle. They were fed *ad libitum* and conditioned in a nonstressful environment for at least 1 week prior to experiments. Experiments were performed in accordance with the Guide for the Care and Use of Laboratory Animals of Huazhong University of Science and Technology and approved by the Ethics Committee.

After 5 weeks of diets high in fat and sucrose (consisting of 58.5% basic diet, 20% sucrose, 10% yolk, 10% lard, 1% cholesterol, and 0.5% sodium deoxycholate (w/w)), the rats, fasted for 12 h, were intraperitoneally injected with the fresh prepared solution of STZ (40 mg/kg body weight, BW) in citrate buffer (pH 4.5, 0.1 M). Five days later, the rats with FBG level above 11.1 mmol/L were considered as diabetic rats and still fed with high fat and sucrose diets for 10 days to obtain steady diabetic animal model [28].

Then, the experimental rats were randomly divided into 8 groups with 8 rats each at least—normal control: received 0.3% CMC-Na solution (0.1 mL/10 g, as vehicle) with 10 rats, diabetic control: treated with vehicle with 8 rats, metformin: diabetic rats treated with positive metformin (150 mg/kg BW) with 9 rats, ETH group: diabetic rats received ETH extract (400 mg/kg BW and 200 mg/kg BW) with 9 rats, respectively, DTH group: diabetic rats received DTH extract (400 mg/kg BW and 200 mg/kg BW) with 8 rats, respectively, and corymbiferin group: diabetic rats treated with corymbiferin (40 mg/kg BW) with 9 rats. The doses were equivalent to crude medicine amount when calculated according to the traditional dose for human (9–16 g/day). The FBG level was measured using one touch glucometer (Bayer, Contour TS, Germany) after 5 weeks of treatment. Diet and water were fed *ad libitum*.

### 2.6. Oral Glucose Tolerance Test (OGTT)

On the day before rats were sacrificed, oral glucose tolerance test (OGTT) was performed in 12 h fasted diabetic rats by feeding glucose (2.5 g/kg). The administration of ETH and DTH extracts was orally administrated 60 min before glucose administration. The FBG levels were measured at 0, 60, 120, and 180 min after a gavage of glucose.

### 2.7. Assay of Lipids and Insulin Levels of Blood Samples

At the end of oral administration, the rats were deprived of foods overnight for 12 h. All blood samples were obtained from hearts of the anesthetized rats and centrifuged at 3500 rpm for 15 min to obtain serum samples which were used to detect serum total cholesterol (TC), triglycerides (TG), low-density lipoprotein (LDL), high-density lipoprotein (HDL), and insulin level. TC, TG, LDL, and HDL were measured with Roche Modular analytics P800 (Roche, Switzerland). The insulin levels were determined using Roche Modular analytics E170 (Roche, Switzerland).

### 2.8. Assay of Antioxidant Activity

Once the blood had been collected, the liver was excised, weighed, and washed in ice-cold saline to remove the blood. Then, the liver was homogenized with manual homogenizer to obtain 10% liver homogenate and centrifuged at 4000 rpm for 15 min at 4°C. The supernatant was used for the assay of malondialdehyde (MDA), superoxide dismutase (SOD), catalase (CAT), glutathione peroxidase (GPx), and glutathione (GSH) according to the commercial kits (Jiancheng, Nanjing, China). 

### 2.9. Assay of Glycogen, Glucokinase (GK), and Glucose-6-Phosphatase (G6Pase) Activities

The glycogen content in the liver was assayed according to the methods described in the instructions (Jiancheng, Nanjing, China). The absorbance was read by a Cary 100 spectrophotometer (Varian Medical Systems, USA) at 620 nm and expressed as mg/g wet weight. GK and G6Pase activities in liver homogenate obtained above were estimated by ELISA. The procedure was performed strictly in accordance with the manufacturer's instructions (R&D, USA).

### 2.10. Histological Study

After animals were euthanized, livers and pancreases were collected and put into 10% neutral buffered formalin for 48 h. Then, organs were embedded within paraffin. Solid sections of 4 *μ*m thickness were made using a rotary microtome. The sections were stained with haematoxylin and eosin (H&E) and then observed by light microscopy for histopathological examination (Olympus CX31, Japan).

### 2.11. Western Blot of IRS-2, PI3K, and AKT2 in Livers

The rest liver tissues were stored at −80°C until time of use. The concentrations of total protein extracts from liver tissues were quantified by the BCA assay protein kit (Beyotime, China). Liver tissues (50 mg) were homogenized, and the supernatant obtained by centrifugation was fractionated by electrophoresis on 10% SDS polyacrylamide gels. The fractionated proteins were transferred onto nitrocellulose membrane (Bio-Rad Laboratories, USA) [29, 30] in transfer buffer. The membranes were blocked for 1 h with 5% skim milk in phosphate buffered saline containing 0.05% Tween 20 (PBST) and then incubated, respectively, with primary antibodies against IRS-2, PI3K, and AKT2 (Abcam, USA) overnight at 4°C. Glyceraldehyde-3-phosphate dehydrogenase (GAPDH) was used as a loading control. The membranes were washed and incubated with horseradish peroxidase (HRP) conjugated secondary antibodies IgG or anti-mouse IgG secondary antibody (Amersham Biosciences, USA) for 1 h at room temperature. After the membranes were washed again as above, immunodetection was performed according to the ECL western blotting protocol of Amersham Buchler (Braunschweig, Germany) and quantified by Image-Pro Plus (version 5.0 Media Cybernetics). Experiments were repeated at least twice. 

### 2.12. Acute Toxicity Study

Acute (24 h) oral toxicity study was performed to know whether *S. bimaculata* had any toxic effects. Kunming mice (20 ± 2 g) were divided into groups of 10 mice, and each group included 5 females and 5 males. They were orally administrated with ETH extract by a single dose of 0.47, 1.02, 2.19, 4.65, and 10.00 g/kg. All external morphological, behavioural, and neurological changes and death were recorded continuously [31].

### 2.13. Statistical Analysis

All data were expressed as means ± S.E.M. The statistical significance among multiple groups was assessed by one-way ANOVA using SPSS program. *P* value < 0.05 was considered to be statistically significant.

## 3. Results

### 3.1. HPLC Analysis of ETH and DTH Extracts

**Figure 1:**
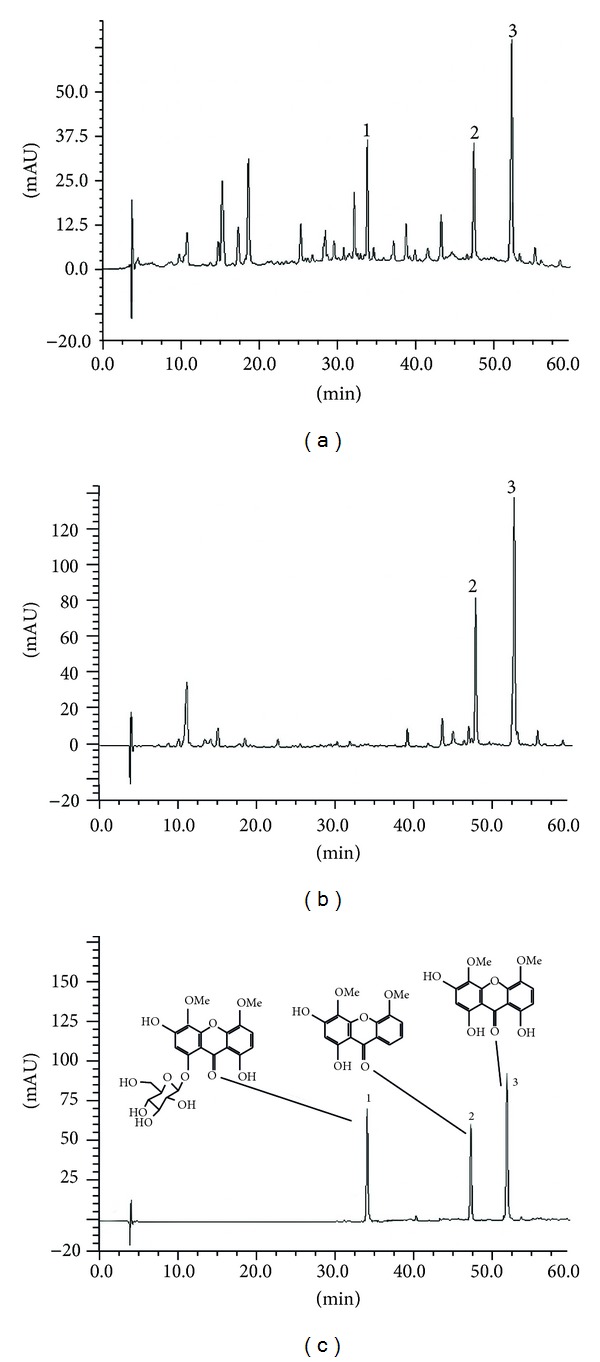
HPLC chromatograms at 254 nm of *S. bimaculata *(A-ETH, B-DTH, C-standard mixture): (1) 1-*O*-glucosylcorymbiferin, (2) 1,3-dihydroxy-4,5-dimethoxyxanthone, and (3) corymbiferin. The HPLC profiles were analyzed with an autosampler and a DAD detector at 0.8 mL/min using gradient elute as follows: solvent methanol and solvent formic acid aqueous (0.1%).

Three compounds in ETH and DTH extracts had been obtained and identified as 1-*O*-glucosylcorymbiferin (1), 1,3-dihydroxy-4,5-dimethoxyxanthone (2), and corymbiferin (3), which were the main constituents in ETH and DTH extracts. The compounds 1, 2, and 3 were quantified as 9.28, 13.19, and 25.44 mg/g, respectively, in ETH extract and 3 as 111.34 mg/g in DTH extract (Figure 1).

### 3.2. Effects of Extracts on Glucose Consumption in 3T3-L1 Cells

**Figure 2:**
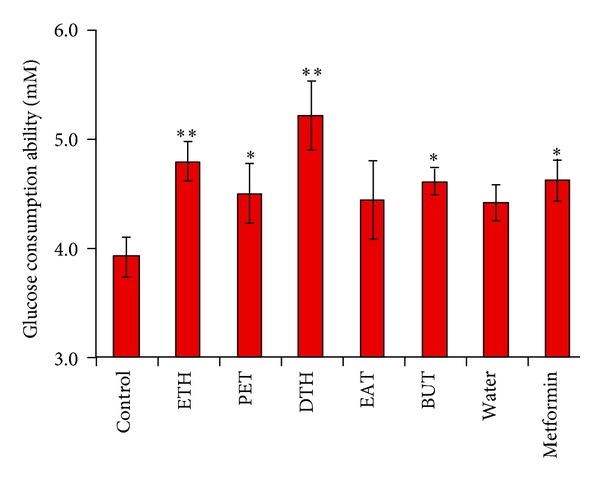
Effect of different extracts (25 *μ*g/mL, resp.) of *S. bimaculata* on glucose consumption in 3T3-L1 adipocyte. Metformin was at 50 *μ*g/mL as positive control. Each value was represented as mean ± S.E.M (*n* = 4). *P < 0.05, **P < 0.01 compared to diabetic control.

The results of the glucose consumption ability of ETH, PET, DTH, EAT, BUT, and water extracts of *S. bimaculata* in 3T3-L1 cells were shown in Figure 2. ETH, PET, DTH, and BUT extracts showed significant activities in increasing the glucose consumption in 3T3-L1 cells at the dose of 25 *μ*g/mL. The rates were increased by 22.45%, 20.41%, 33.16%, and 17.86% (*P* < 0.05 or *P* < 0.01), respectively. In the same condition, the rate of glucose consumption was elevated by 18.11% (*P* < 0.05) in the the presence of metformin at 50 *μ*g/mL. EAT and water extracts had no significance (*P* > 0.05) in increase of glucose consumption. It is obvious that the ETH and DTH extracts had a better glucose-lowering effect (*P* < 0.01) than the other extracts and metformin. Therefore, ETH and DTH extracts were further evaluated for their antidiabetic activity *in vivo* model.

### 3.3. Effects of ETH and DTH Extracts and Corymbiferin on FBG in Diabetic Rats

The FBG level measured in normal and diabetic rats on days 0, 7, 14, 25, and 35 of treatment was shown in Table 1. STZ-induced diabetic rats showed significant increase in blood glucose levels compared with normal rats. After administration of DTH extract at 400 mg/kg and 200 mg/kg for 5 weeks, the FBG level was significantly decreased (*P* < 0.05 or *P* < 0.01) when compared to diabetic rats. The ETH extract (400 mg/kg and 200 mg/kg) showed less significant effect on the FBG levels. The treatment with corymbiferin (40 mg/kg) also reduced the FBG level significantly (*P* < 0.05 or *P* < 0.01), similar to the effect of positive metformin on the diabetic rats.

### 3.4. Effects of ETH and DTH Extracts and Corymbiferin on OGTT in Diabetic Rats

**Figure 3:**
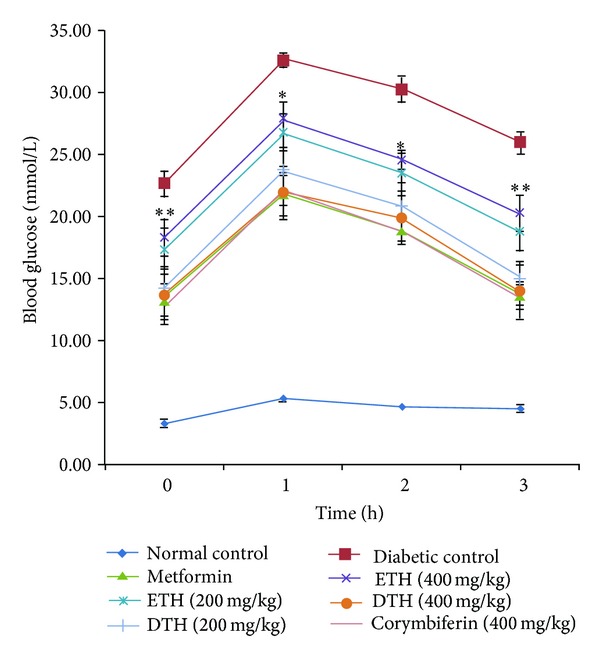
Effects of *S. bimaculata* on oral glucose tolerance in diabetic rats. The blood glucose levels were measured at 0, 60, 120, and 180 min after a gavage of sucrose. Data are shown as means ± S.E.M. *P < 0.05, **P < 0.01 compared to diabetic group.

As shown in Figure 3, the oral glucose tolerance of diabetic rats was severely impaired compared to normal rats. After treatment, it was observed that the glucose tolerance abilities were improved from the 1st to 3rd hour. Compared to diabetic control, DTH extract treated group showed significant hypoglycemia and steady declines (*P* < 0.01) from 1st to 3rd hour. The ETH extract showed less significant effect on the FBG levels. Furthermore, corymbiferin also significantly reduced the blood glucose and showed a similar effect to metformin on OGTT.

### 3.5. Effects of ETH and DTH Extracts and Corymbiferin on Serum Lipid Profiles and Insulin Level in Diabetic Rats

As shown in Table 2, in diabetic rats, the TC, TG, and LDL levels were significantly increased (*P* < 0.05 or *P* < 0.01). After being treated by ETH and DTH extracts and corymbiferin, the increased serum levels of TC, TG, and LDL were significantly suppressed (*P* < 0.05 or *P* < 0.01) in diabetic rats. Among these lipid parameters, the LDL levels declined to near normal status in the treated groups. DTH extract and corymbiferin showed more remarkable TC-lowering and LDL-lowering activities, better than the positive control. The HDL level was not significantly elevated in the treated groups, but HDL/LDL ratio had significant differences after treatment. Meanwhile, there was a significant increase in insulin levels (*P* < 0.05) in DTH extract, corymbiferin, and metformin treated groups compared to the diabetic control. The ETH extract showed no significant effect on insulin level (*P* > 0.05).

### 3.6. Effects of ETH and DTH Extracts and Corymbiferin on Antioxidant Status in Diabetic Rats

Oxidative stress assessment was performed by recording the activities of antioxidative enzymes and peroxidation. As shown in Table 3, a significant increase in MDA level and a significant decrease in CAT, SOD, GPx, and GSH activities were observed in diabetic control. After the treatment with ETH and DTH extracts and corymbiferin, MDA levels were significantly decreased (*P* < 0.01) to near normal status, while CAT, SOD, GPx, and GSH activities were significantly increased (*P* < 0.05 or *P* < 0.01, resp.) compared to diabetic control. Moreover, corymbiferin showed the best activity of decreasing lipid peroxidation.

### 3.7. Effects of ETH and DTH Extracts and Corymbiferin on GK and G6Pase Activities, and Glycogen Content in Liver of Diabetic Rats

The content of glycogen and the activities of GK and G6Pase in the liver of diabetic rats were represented in Table 4. Compared with diabetic control, both ETH and DTH extracts significantly decreased G6Pase activity while increased glycogen content and GK activity, and DTH extract was superior to (*P* < 0.05 or *P* < 0.01) ETH extract. In corymbiferin treated group, glycogen content and GK activity were higher while G6Pase activity was lower than in the other groups.

### 3.8. Histological Results

**Figure 4:**
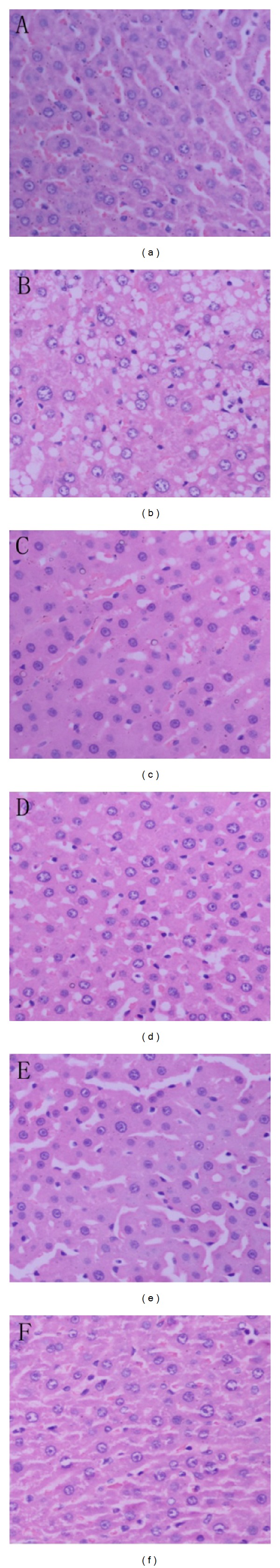
Effects of *S. bimaculata* on liver damage in diabetic rats. (a) Normal control; (b) diabetic control; (c) metformin; (d) ETH; (e) DTH; (f) corymbiferin. The livers were sectioned and stained with haematoxylin and eosin (H&E, magnification 400x) after being fixed and embedded.

**Figure 5:**
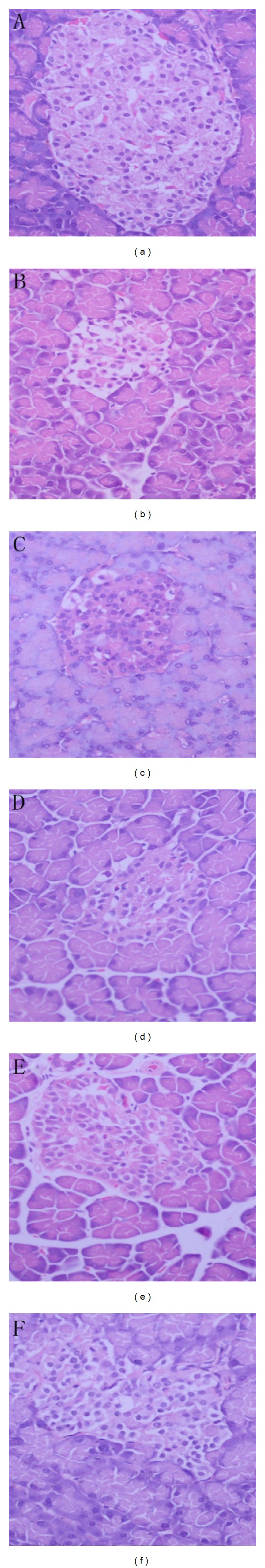
Effects of *S. bimaculata* on pancreatic damage in diabetic rats. (a) Normal control; (b) diabetic control; (c) metformin; (d) ETH; (e) DTH; (f) corymbiferin. The pancreases were sectioned and stained with haematoxylin and eosin (H&E, magnification 400x) after being fixed and embedded.

As shown in Figure 4, clearly adipose accumulation and fat vacuoles were observed in diabetic control. After treatment, the above abnormal changes were significantly reversed. In the study, hepatic lobular structures seemed to show little degeneration, which were remarkably improved by ETH and DTH extracts and corymbiferin treatment. Meanwhile, histopathological analyses revealed that STZ caused severe injury to the pancreas (Figure 5). In diabetic rats, abnormal changes were evident in the form of an obvious reduction of pancreatic islets, irregular shape, and atrophic changes, which were ameliorated after 35 days of treatment. However, ETH extract did not markedly improve the histopathology of *β*-cells.

### 3.9. Effect of ETH and DTH Extracts and Corymbiferin on IRS-2/PI3K/AKT2 Insulin Signaling Pathway

**Figure 6:**
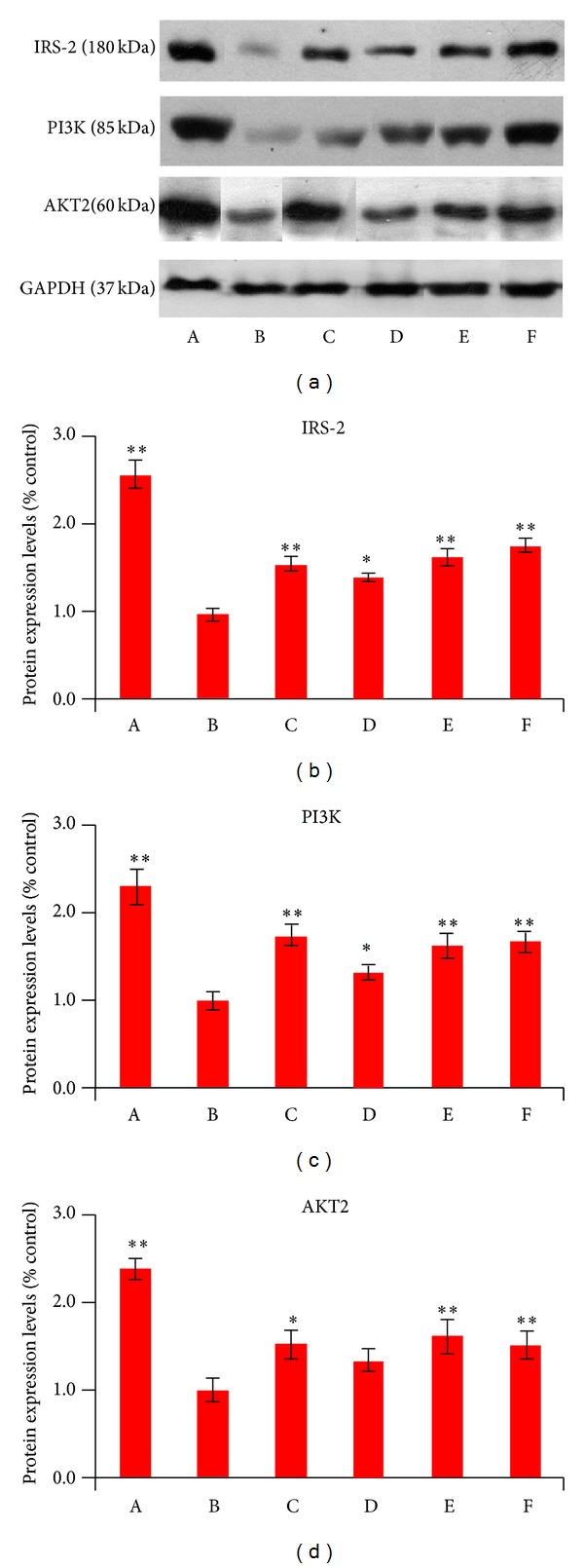
Effect of *S. bimaculata* on protein expression of IRS-2, PI3K, and AKT2 in liver tissues of diabetic rats. (a) Representative radiographs of IRS-2, PI3K, and AKT2. (b) Quantitative analysis of western blot. A: normal control; B: diabetic control; C: metformin; D: ETH; E: DTH; F: corymbiferin. Data were shown as the means ± S.E.M. *P < 0.05, **P < 0.01 compared to diabetic group.

The protein expression levels of IRS-2, PI3K, and AKT2 in liver tissues of diabetic rats were investigated by western blot (Figure 6). The results indicated that the levels of IRS-2, PI3K, and AKT2 in the liver of diabetic rats were remarkably diminished when compared to the normal rats (*P* < 0.01). After treatment, the expression of IRS-2, PI3K, and AKT2 in diabetic rats was found to increase significantly. In particular, the protein levels of IRS-2, PI3K, and AKT2 were more remarkably expressed (*P* < 0.01) in DTH and corymbiferin treated groups. Thus, western blot results showed that DTH and corymbiferin could improve insulin signaling transduction of diabetic rats, which resulted in the improvement of insulin sensitivity.

### 3.10. Acute Toxicity Studies

All animals showed good tolerance to testing given single doses of ETH extract. Animals did not show noticeable signs of toxic effects on behavior or appearance, and all mice survived during the whole experimental period. The body weight and food consumption were normal when compared to vehicle treated mice.

## 4. Discussions

*S. bimaculata* plant has long been used as a folk medicine for treating hepatitis, gastroenteritis, and urinary tract infection. The current study was designed to evaluate the effects of different extracts on glucose consumption in 3T3-L1 cells to screen the optimum antidiabetic extracts, of which the chemical components were analyzed by HPLC. Then, the active extracts and their major compound corymbiferin were further studied in STZ-induced diabetic rats *in vivo*. The hyperglycemic activity, antioxidant effects, and antidiabetic mechanisms accompanied with the antidiabetic substances of *S. bimaculata* were elucidated.

The results in 3T3-L1 cell assay on glucose consumption demonstrated that ETH, PET, DTH, EAT, BUT, and water extracts could increase the glucose consumption. ETH and DTH extracts showed better glucose-lowering effect in 3T3-L1 cell line than the other extracts and metformin. It is suggested that the two extracts could increase the glucose utilization in adipose tissue. From HPLC analysis, it was found that tetra- and pentaoxygenated xanthones were the main chemical constituents of ETH and DTH extracts. Among the xanthones, the corymbiferin was the most abundant one, quantified to be 2.5% in ETH extract and 11.1% in DTH extracts, respectively. So ETH and DTH extracts and corymbiferin were selected for *in vivo* high fat fed and low-dose STZ-induced diabetic rats. 

High fat and sucrose diet and low-dose STZ have been known to induce a mild impairment of insulin secretion which is similar to the feature of type 2 diabetes [32–34]. In the present study, after being treated by ETH and DTH extracts for 35 days, there were a decreasing blood glucose level and simultaneous increasing serum insulin level. It was found that DTH extract showed relatively more significant effects. Further observation was that corymbiferin also showed more significant effects on decreasing blood glucose and increasing insulin levels, similar to the effect of the positive metformin. The histopathology results indicated the obvious improvement and protection of pancreatic *β*-cells by DTH extract and corymbiferin, suggesting that the decreased blood glucose and increased insulin concentration are due to the relatively sufficient insulin secretion from the remaining pancreatic *β*-cells. 

 It is believed that the PI3K/AKT pathway is considered to be the major effector of metabolic insulin action [35–37]. Deregulation of the phosphoinositide-3-kinase (PI3K) and v-akt murine thymoma viral oncogene homolog (AKT), which are essential for glucose homeostasis, often results in obesity and diabetes [36]. Therefore, the expressions of IRS2, PI3K, and AKT2 in liver tissues of experimental rats were investigated in this present study. The results revealed that the two extracts and corymbiferin enhanced the activity and expressions of IRS2, PI3K, and AKT2 which were initially downregulated in hyperglycemic rats. The DTH extract and corymbiferin showed relatively more remarkable effects on IRS2, PI3K, and AKT2 activation. This suggested that DTH extract and corymbiferin could obviously improve insulin signaling transduction of diabetic rats, which resulted in the improvement of insulin sensitivity. 

In the liver, insulin blocks the release and neogenesis of glucose and stimulates glucose storage. The hepatic glucose metabolism is regulated by the activities of key enzymes of glucokinase, glucose-6-phosphatase, fructose 1 and 6-bisphosphatase [38, 39]. In the present study, the liver glycogen content was remarkably reduced in diabetic rats. The treatment with ETH and DTH extracts and corymbiferin brought back hepatic glycogen level. Moreover, increased GK activity and decreased G6Pase activity in diabetic rats were also observed after treatment. On further analysis, it was still found that DTH extract and corymbiferin showed superior effects on the increase of glycogen synthesis and GK activity and decrease of G6Pase activity, which could be partially due to increased insulin release [40].

The abnormality of lipid metabolism associated with diabetes mellitus may be due to insulin deficiency [41]. Insulin deficiency inactivates lipoprotein lipase which hydrolyses triglycerides and prevents mobilization of free fatty acid in normal state, thereby resulting in phospholipid level [42, 43]. After the long-term administration of DTH extract and corymbiferin, obviously lowered serum TC, TG, and LDL levels along with raised HDL/LDL ratio in diabetic rats were observed. The strong activities in the modulation of lipid metabolism implied that DTH and corymbiferin of *S. bimaculata* could be more beneficial for preventing cardiovascular complications of diabetes mellitus such as ischemic heart disease [44] and the formation of atherosclerosis [45]. 

Lines of evidence support that hyperglycemia induces the overproduction of oxidative stress [46]. The imbalance between oxidative stress and antioxidative defense in diabetics may result in cell and tissue damage, which plays an important role in the pathogenesis of diabetic complications. SOD, CAT, GPx (the enzymatic antioxidants), and GSH (a nonenzymatic antioxidant) work synergistically and in combination with each other to protect the body against oxidative damage [47]. In this study, significant increases of SOD, CAT, and GPx activities accompanied with obvious increase of GSH level in ETH and DTH extracts and corymbiferin-treated groups were observed, suggesting that they had strong antioxidative activities. MDA reflects the degree of lipid peroxidation which is one of the characteristic features of diabetes mellitus [48, 49]. After being treated, MDA levels were dramatically reduced towards near normal status by ETH and DTH extracts and corymbiferin, which thereby prevented the tissue from injury of lipid peroxidation and hyperglycemia. Therefore, these mutual effects clearly showed that ETH and DTH extracts and corymbiferin are beneficial for attenuating diabetes mellitus through inducing IRS serine/threonine phosphorylationthe, since overproduction of ROS can impair insulin signaling caused by oxidative stress in diabetic rats [2, 50].

As the main component of DTH extract, corymbiferin may take responsibility for hypoglycemia and enhancement of insulin sensitivity due to its bases on 1,3,5- or 1,3,8-oxygenated systems, similar to bellidifolin and swerchirin that have been demonstrated to have blood-glucose-lowering effect in diabetic animals through enhancing insulin signaling transduction [16–19]. And also corymbiferin, belonging to polyphenol compounds, could contribute to improving diabetes mellitus and preventing the formation of diabetic complications due to its free radical scavenging properties. Therefore, corymbiferin in ETH or DTH extract could be one lead compound for the amelioration of hyperglycemia.

In conclusion, the antidiabetic activity of *S. bimaculata *is well established. One potent mechanism attributes to the protection of pancreatic *β*-cells and liver tissues by ameliorating lipid metabolism and oxidative stress. The other mechanism is also associated with amelioration of carbohydrate metabolism by enhancing insulin signaling and regulating the rate-limiting enzymes. The xanthone(s) like corymbiferin of *S. bimaculata* was partially responsible for its antidiabetic effect. However, further effort is required to demonstrate the other underlying mechanisms.

## Figures and Tables

**Table 1:** Effects on blood glucose levels in diabetic rats.

Groups	Blood glucose levels (mmol/L)				
	Initial	On the 7th day	On the 14th day	On the 25th day	On the 35th day
Normal	03.30 ± 0.13**	03.10 ± 0.13**	03.26 ± 0.29**	03.78 ± 0.17**	03.28 ± 0.14**
Diabetic	21.32 ± 1.26	23.96 ± 1.19	25.56 ± 2.31	23.86 ± 1.28	22.64 ± 1.02
Metformin	22.73 ± 2.22	22.83 ± 1.85	22.08 ± 2.02	14.45 ± 1.45**	13.22 ± 1.86**
ETH (400 mg/kg)	21.26 ± 0.86	22.91 ± 1.65	23.66 ± 1.53	22.13 ± 0.98	18.25 ± 1.51
ETH (200 mg/kg)	20.81 ± 0.99	24.00 ± 1.15	21.59 ± 1.20	20.83 ± 1.24	17.25 ± 1.87*
DTH (400 mg/kg)	20.49 ± 0.76	24.91 ± 0.87	19.80 ± 1.69*	15.53 ± 1.69**	13.61 ± 2.28**
DTH (200 mg/kg)	20.78 ± 0.84	24.24 ± 1.47	20.49 ± 1.51*	15.99 ± 1.44**	14.26 ± 1.45**
Corymbiferin	21.32 ± 0.68	26.14 ± 0.93	19.56 ± 1.53*	14.87 ± 1.54**	12.26 ± 1.15**

Data were shown as the means ± S.E.M. *P < 0.05, **P < 0.01 compared to diabetic group.

**Table 2:** Effects on serum lipid and insulin levels in diabetic rats.

Groups	TC (mmol/L)	TG (mmol/L)	LDL (mmol/L)	HDL/LDL	Insulin (mU/L)
Normal	1.70 ± 0.07**	0.79 ± 0.08*	0.24 ± 0.02	6.80 ± 0.52	7.81 ± 0.28*
Diabetic	2.42 ± 0.07	1.35 ± 0.15	0.45 ± 0.02	4.46 ± 0.20	3.97 ± 0.14
Metformin	2.27 ± 0.14	0.99 ± 0.18	0.31 ± 0.03**	6.79 ± 0.88	4.86 ± 0.17*
ETH (400 mg/kg)	2.31 ± 0.12	1.02 ± 0.20	0.32 ± 0.03**	7.35 ± 0.65	4.2 ± 0.19
ETH (200 mg/kg)	1.91 ± 0.12*	0.84 ± 0.08*	0.24 ± 0.02**	8.03 ± 0.80*	4.47 ± 0.20
DTH (400 mg/kg)	2.00 ± 0.15	0.59 ± 0.10**	0.26 ± 0.08**	7.72 ± 0.76*	4.80 ± 0.24*
DTH (200 mg/kg)	1.72 ± 0.09**	0.51 ± 0.06**	0.20 ± 0.01**	8.28 ± 0.31**	4.69 ± 0.18*
Corymbiferin	1.96 ± 0.16*	0.56 ± 0.06**	0.24 ± 0.03**	9.29 ± 1.26**	4.81 ± 0.31*

Data were shown as the means ± S.E.M. *P < 0.05, **P < 0.01 compared to diabetic group.

**Table 3:** Effects on CAT, GSH, GPx, SOD, and MDA from liver tissues.

Groups	CAT (U/mg protein)	GSH (nmol/mg protein)	GPx (unit)	SOD (U/mg protein)	MDA (nmol/mg protein)
Normal	40.32 ± 3.30**	412.49 ± 20.03**	399.21 ± 22.33**	166.59 ± 16.50**	1.28 ± 0.37**
Diabetic	16.59 ± 4.05	246.40 ± 6.73	292.34 ± 23.71	117.56 ± 6.84	2.80 ± 0.80
Metformin	33.64 ± 6.00**	326.64 ± 11.52**	336.30 ± 15.70	140.71 ± 5.47**	1.49 ± 0.27**
ETH (400 mg/kg)	24.01 ± 6.29	337.69 ± 9.23**	295.38 ± 9.04	133.03 ± 7.48**	1.46 ± 0.33**
ETH (200 mg/kg)	26.39 ± 4.78**	357.73 ± 11.85**	340.31 ± 27.01	141.14 ± 4.83**	1.36 ± 0.19**
DTH (400 mg/kg)	28.32 ± 5.57**	369.24 ± 18.23**	352.80 ± 8.12*	138.49 ± 12.48**	1.11 ± 0.15**
DTH (200 mg/kg)	25.91 ± 5.65**	366.05 ± 25.18**	320.17 ± 12.72	126.76 ± 11.25	1.22 ± 0.21**
Corymbiferin	26.45 ± 5.20**	297.66 ± 4.63*	431.88 ± 13.36**	136.67 ± 16.30**	1.06 ± 0.10**

Data are shown as the means ± S.E.M. *P < 0.05, **P < 0.01 compared to diabetic group.

**Table 4:** Effects on glycogen, GK, and G6Pase in diabetic rats.

Groups	Liver		
	Glycogen (mg/g tissue)	GK (mU/mg protein)	G6Pase (mU/mg protein)
Normal	8.60 ± 2.15**	106.30 ± 7.67**	365.67 ± 24.89**
Diabetic	4.82 ± 1.13	81.12 ± 9.47	454.15 ± 64.81
Metformin	7.32 ± 1.25*	95.23 ± 7.31**	388.00.±43.64*
ETH (400 mg/kg)	6.15 ± 0.85	90.94 ± 6.40	401.54 ± 61.32
ETH (200 mg/kg)	6.85 ± 1.82*	89.58 ± 11.50	391.59 ± 40.67*
DTH (400 mg/kg)	7.21 ± 2.41*	94.95 ± 7.80**	378.93 ± 55.13**
DTH (200 mg/kg)	7.06 ± 1.19*	94.00 ± 9.22*	379.47 ± 55.39**
Corymbiferin	7.59 ± 3.25**	104.27 ± 10.18**	381.75 ± 54.09**

Data were shown as the means ± S.E.M. *P < 0.05, **P < 0.01 compared to diabetic group.
